# Supplementary material for: Hydrophobic Recovery of PDMS Surfaces in Contact with Hydrophilic Entities: Relevance to Biomedical Devices
Source: Materials (Basel). 2022 Mar 21;15(6):2313. doi: 10.3390/ma15062313 (PMC8950181; doi:10.3390/ma15062313)
Supplement: Supplementary file 1 [file materials-15-02313-s001.zip › materials-1613774-supplementary.pdf]

Hydrophobic recovery of PDMS surfaces in the contact with hydrophilic entities: relevance to biomedical applications

Tomoo Tsuzuki, Karine Baassiri, Zahra Mahmoudi, Ayyappasamy Sudalaiyadum Perumal, Kavya Rajendran, Gala Montiel Rubies, Dan V. Nicolau

Supplementary Information

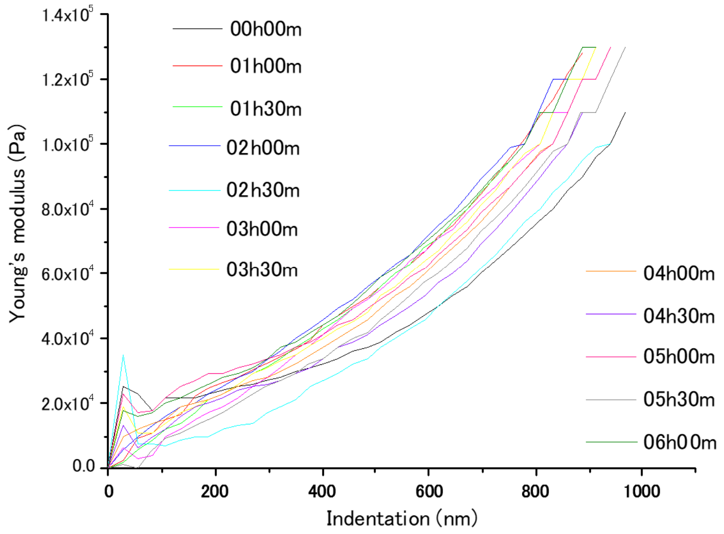

Figure S1. Estimated Young's modulus for AFM F-d measurements for various stages of hydrophobic recovery.

Table S1. Assignment of the FTIR peaks in Figure 3.

Peak	IR region (cm^{-1})	Description
1	785–815	$-CH_3$ rocking and ≡Si–C≡ stretching in $\equiv Si-CH_3$
2	825–865	≡Si–O stretching in ≡Si–OH
3	875–920	≡Si–O stretching in ≡Si–OH
4	1015–1150	In-phase and out-of-phase wagging vibrations of $-(CH_2)-$ in $\equiv Si-(CH_2)_2-Si\equiv$ and $\equiv Si-CH_2-Si\equiv$
5	1055–1090	Asymmetric ≡Si–O–Si≡ stretching in $[-(CH_2)_2Si-O-]_x$
6	1100–1170	In-phase and out-of-phase wagging vibrations of $-(CH_2)-$ in $\equiv Si-(CH_2)_2-Si\equiv$
7	1245–1270	Symmetric $-CH_3$ deformation in $\equiv Si-CH_3$
8	2950–2970	Asymmetric $-CH_3$ stretching in $\equiv Si-CH_3$
9	3050–3700	–OH stretching in ≡Si–OH, possibly also in ≡C–OH (3610–3640 cm^{-1})

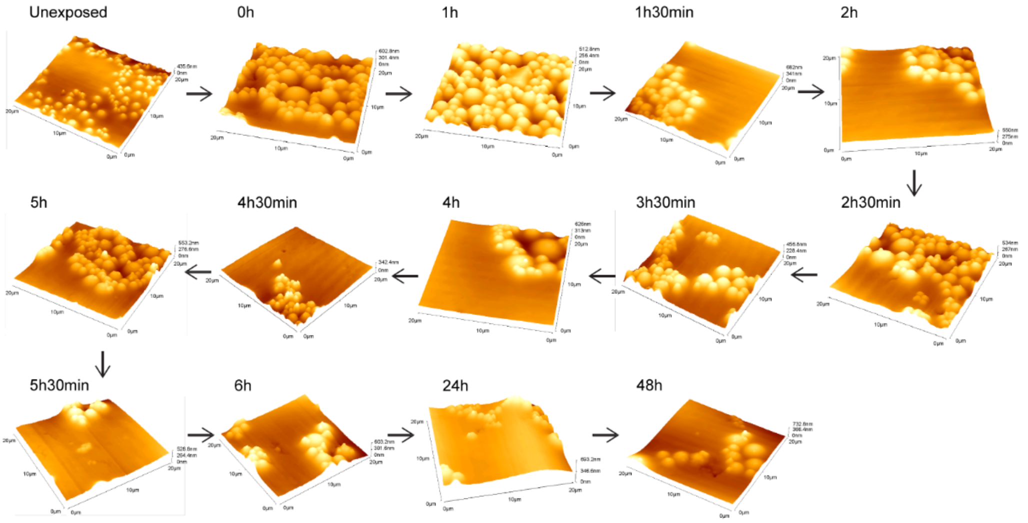

Figure S2. Sequence of AFM scans of the topography of PDMS embedded with beads, unexposed, immediately after UV exposure, and at various stages of hydrophobic recovery.

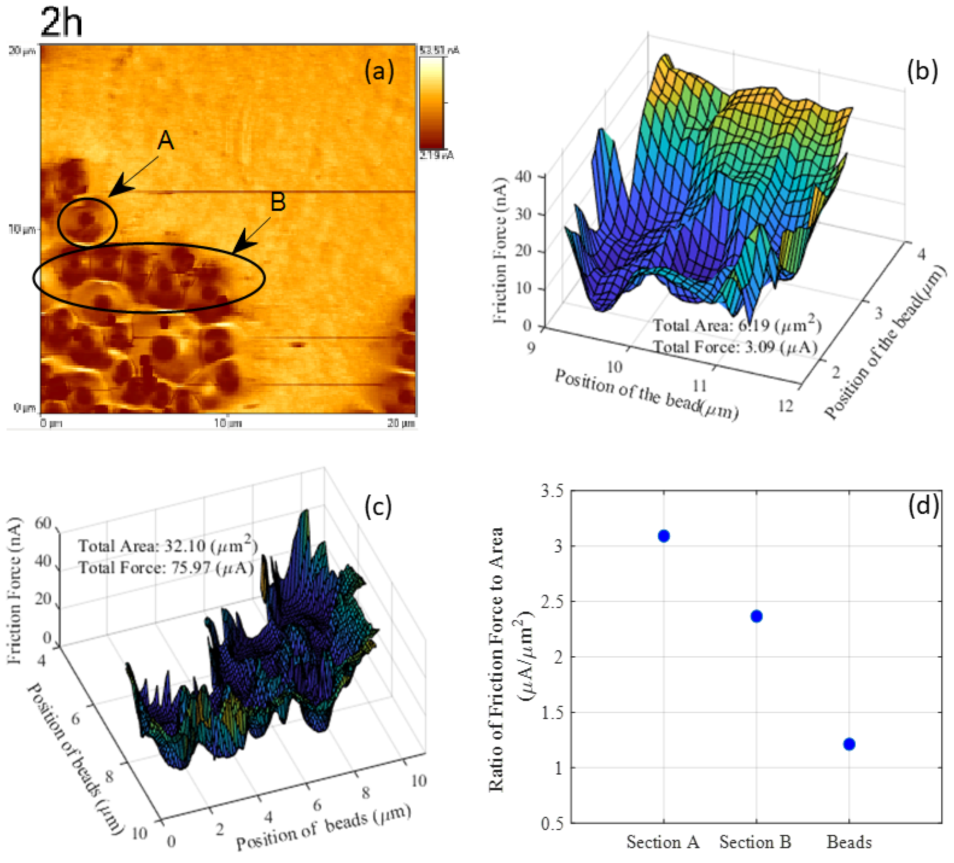

Figure S3. (a) AFM lateral force scans of PDMS surfaces with embedded hydrophilic beads, (b, c) friction forces on the beads and surrounding area in A and B section (d) ratio of friction forces to the area of beads and their vicinity.
